# The relationship between the severity of depression in postmenopausal women and the incidence of osteoporosis and subsequent fractures: A cross-sectional observational study

**DOI:** 10.1097/MD.0000000000047750

**Published:** 2026-02-28

**Authors:** Yichao Qiu, Jiande Chen, Xiaoqin Qian, Haifang Sun, Lina Yu, Xiang Chen

**Affiliations:** a Women’s Health Department, Shaoxing Keqiao Women & Children’s Hospital, Shaoxing, Zhejiang, China; b Spine (Bone Tumors) Department, Shaoxing Municipal Hospital of Traditional Chinese Medicine, Shaoxing, Zhejiang, China; c Laboratory Department, Shaoxing Keqiao Women&Children’s Hospital, Shaoxing, Zhejiang, China.

**Keywords:** depression, osteoporotic fracture, postmenopausal osteoporosis, postmenopausal women

## Abstract

Postmenopausal women experience declining estrogen levels that create a close association between the incidence of osteoporosis and osteoporotic fractures and the severity of depression, with recent evidence suggesting that depression may further exacerbate bone loss and fracture risk through influences on bone metabolism, lifestyle factors, and medication adherence. To evaluate the relationship between depression severity, bone mineral density, and fracture events, we conducted a combined cross-sectional and prospective cohort study of 245 postmenopausal women. Depression severity was measured using the HAMD-17 scale, bone mineral density was assessed by dual-energy X-ray absorptiometry, and fracture events were confirmed radiographically. Participants were stratified by age, menopause age, body mass index, smoking history, previous fracture history, and comorbidities to assess the impact of depression severity on osteoporosis and fracture risk. Our results demonstrated that depression severity showed significant inverse correlations with lumbar spine bone mineral density (odds ratio = 0.55, 95% confidence interval: 0.40–0.76, *P* < .001) and hip bone mineral density (odds ratio = 0.68, 95% confidence interval: 0.52–0.89, *P* < .005), and was positively associated with the incidence of osteoporotic fractures (odds ratio = 3.42, 95% confidence interval: 2.58–4.53, *P* < .001). Multivariate logistic regression confirmed that depression severity was independently associated with osteoporosis (odds ratio = 2.85, 95% confidence interval: 2.12–3.82, *P* < .001), while Cox regression analysis showed that it significantly increased fracture risk (hazard ratio = 2.90, 95% confidence interval: 2.10–4.00, *P* < .001). Subgroup analyses revealed more pronounced associations in women with longer postmenopausal duration exceeding 5 years, lower body mass index below 24 kg/m^2^, and those with comorbid diabetes. These findings indicate that depression severity in postmenopausal women is significantly associated with reduced bone density, increased prevalence of osteoporosis, and higher fracture rates, offering new strategies for managing the comorbidity of depression and osteoporosis in this population and highlighting the necessity for integrated mental and skeletal health interventions.

## 1. Introduction

Menopause represents a critical transitional period in a woman’s life, marking the cessation of ovarian function and a precipitous decline in estrogen levels.^[1]^ This physiological shift triggers a wide range of health concerns, among which osteoporosis and its associated fragility fractures constitute one of the most severe and costly long-term consequences.^[2]^ OP is a prevalent skeletal disorder characterized by low bone mass and deterioration of bone tissue microarchitecture, leading to increased bone fragility and a high risk of fracture following minor trauma or even daily activities such as bending or coughing.^[3]^ OF also termed fragility fractures, are defined as fractures resulting from low-energy trauma, equivalent to a fall from standing height or less.^[4]^ They represent the most serious outcome of OP and are closely related to reduced bone mass. Hip, vertebral, and wrist fractures are the most common osteoporotic complications. These fractures not only cause acute pain, disability, and a severe decline in quality of life but are also significantly associated with substantial morbidity and mortality, imposing a heavy economic burden on global healthcare systems.^[5]^

Concurrently, the perimenopausal and postmenopausal periods are also a high-risk time for the onset of affective disorders, particularly depression, in women. Fluctuations and the eventual depletion of estrogen are believed to play a key role in this process by affecting neurotransmitters and the function of the hypothalamic-pituitary-adrenal (HPA) axis.^[6]^ Traditionally, osteoporosis and depression have been viewed as 2 independent postmenopausal complications.^[7]^ However, a growing body of epidemiological and clinical evidence indicates a significant and strong association between these 2 conditions. On one hand, chronic pain, functional limitations, and altered body image caused by OP may induce or exacerbate depressive symptoms.^[8]^ On the other hand, and more importantly, depression itself may act as an independent risk factor, directly or indirectly accelerating bone loss and increasing fracture risk.^[9]^

Depression may influence bone metabolism and fracture risk through multiple interrelated biological and behavioral pathways. Biologically, chronic stress and depressive states can lead to sustained activation of the HPA axis, resulting in elevated cortisol levels that directly inhibit osteoblast activity and promote osteoclastogenesis. Concurrently, the chronic low-grade inflammatory state associated with depression – characterized by elevated cytokines such as IL-6 and TNF-α – may further accelerate bone resorption. Additionally, depression is often accompanied by decreased estrogen levels, which further undermines skeletal protection. Behaviorally and clinically, individuals with depression frequently exhibit lifestyle factors detrimental to bone health, including reduced physical activity, limited sun exposure, and poor nutritional intake (e.g., inadequate calcium and vitamin D). Furthermore, depression is strongly linked to impaired medication adherence, which may reduce the effectiveness of prescribed osteoporosis treatments and further elevate fracture risk. Thus, depression may act not only as a psychosocial consequence of osteoporosis and fractures, but also as an independent and modifiable risk factor that exacerbates bone loss through intertwined metabolic, lifestyle, and clinical management pathways.

This link between depression and bone deterioration may be particularly pronounced in postmenopausal women compared to the general older population. While depression is a recognized risk factor for bone loss in aging individuals, the postmenopausal state introduces a critical amplifying factor: the acute and sustained decline in estrogen. Estrogen exerts direct protective effects on bone by inhibiting osteoclast activity and promoting osteoblast survival. Its precipitous loss after menopause creates a baseline state of accelerated bone resorption. When superimposed on this high-turnover state, the additional biological burden of depression – through cortisol-mediated bone suppression, inflammation, and potentially further estrogen reduction – may have a synergistic or additive detrimental effect on skeletal integrity. Furthermore, the menopausal transition itself is a period of heightened vulnerability for new-onset or worsening depression, potentially creating a longer exposure window to this dual risk. Therefore, in postmenopausal women, depression may not merely add to the general age-related fracture risk but may interact with estrogen deficiency to disproportionately accelerate bone loss and elevate fracture incidence, warranting targeted clinical attention.

While the epidemiological association between depression and poor bone health is increasingly recognized, significant gaps persist in the extant literature, which the current study aims to address. First, from an epidemiological and clinical perspective, most prior studies have treated depression as a binary (present/absent) diagnosis or relied on symptom questionnaires without detailed severity stratification. This approach may obscure a potential dose-response relationship between the intensity of depressive symptoms and the degree of bone deterioration or fracture risk – a relationship our study explicitly investigates using the HAMD-17 scale. Second, mechanistic clinical data remain fragmented. Although biological pathways (e.g., HPA axis dysfunction, inflammation) are proposed, comprehensive studies simultaneously measuring depression severity, a panel of bone turnover markers (PINP, β-CTX, N-MID), estradiol levels, and bone mineral density (BMD) in a well-defined postmenopausal cohort are scarce. Consequently, the integrated pathophysiological profile linking depressive symptomatology to specific bone metabolic disturbances in this population is poorly characterized. Third, there is a notable lack of prospective data evaluating whether depression severity is an independent predictor of subsequent osteoporotic fractures in postmenopausal women, after rigorously adjusting for key confounders including baseline BMD. Much of the existing evidence is cross-sectional or derived from general elderly populations, limiting causal inference and specificity to the high-risk postmenopausal demographic. This study is therefore justified as it employs a combined cross-sectional and prospective design to: quantify the association between depression severity and comprehensive bone health indices; assess the independent predictive value of depression severity for both osteoporosis prevalence and incident fractures; and explore effect modifiers through subgroup analysis, thereby providing a more nuanced and clinically actionable evidence base.

Many studies, both domestic and international, have predominantly focused on single risk factors in relation to either OP or OF, and numerous risk factors for postmenopausal OF have indeed been confirmed.^[10]^ However, research comprehensively investigating the combined relationship between the severity of depression and both OP and OF in postmenopausal women remains relatively scarce and lacks substantial clinical data support.

Based on this background, this study aims to systematically explore the association between depression severity and the incidence of OP and subsequent fractures in postmenopausal women. The study will employ standardized depression assessment scales to screen and stratify a postmenopausal cohort, while utilizing BMD measurements and radiographic examinations to confirm diagnoses of OP and fractures. We hypothesize that the severity of depression in postmenopausal women is associated with the prevalence of OP and the incidence of fractures. The specific objectives are: To investigate the correlation between depression severity and skeletal health by analyzing parameters including lumbar spine BMD; bone turnover markers (BTMs): PINP, β-CTX, N-MID; bone metabolism-related indicators: estradiol (E2); and general characteristics such as age and BMI across different depression severity strata. To evaluate the differences in OP prevalence among postmenopausal women stratified by depression severity. Analyze whether depression severity is an independent predictor of OF in postmenopausal women. To assess the impact of depression severity on the risk of developing OP and OF, adjusting for factors including age, age at menopause, BMI, smoking history, previous fracture history, and comorbidities.

The findings of this study will assist clinicians in the earlier identification of postmenopausal women at concurrent risk for both mental health and skeletal health issues. It will provide a solid theoretical foundation for establishing an integrated prevention and management model that combines psychological assessment with bone health management.

## 2. Materials and methods

### 2.1. Study design and participants

This was a cross-sectional observational study conducted at our institution between January 2022 and January 2025. The primary objective was to evaluate the correlation between the severity of depression and the incidence of OP and subsequent OF in postmenopausal women.

Participants were recruited from the perimenopausal clinic and the women and children psychological clinic of our hospital, as well as the outpatient and inpatient departments of Shaoxing City Hospital. The inclusion criteria were: clinical diagnosis of depression according to the Diagnostic and Statistical Manual of Mental Disorders, Fifth Edition (DSM-5) criteria; female sex, aged ≥ 45 years; natural menopause for ≥1 year; and provision of informed consent to participate in the study.

Exclusion criteria included: concomitant diseases severely affecting bone metabolism, such as hyperparathyroidism or rheumatoid arthritis; preexisting diagnosis of OP or long-term use of medications affecting bone metabolism (bisphosphonates, glucocorticoids) prior to enrollment; presence of severe cognitive impairment or inability to complete questionnaires and follow-up assessments; and current participation in other interventional clinical trials.

### 2.2. Data collection

Baseline demographic and clinical data were collected, including age, age at menopause, BMI, smoking history, previous fracture history, and comorbidities, to assess the impact of depression severity on the risk of developing OP and OF in postmenopausal women. Furthermore, the correlation between depression severity and skeletal health was analyzed by examining the following parameters across different depression severity strata: lumbar spine BMD; BTMs: PINP, β-CTX, N-MID; bone metabolism-related indicators: E2; and general characteristics including age and BMI.

Depression severity was assessed using the Hamilton depression rating scale (HAMD-17), and patients were categorized into no, mild, moderate, and severe depression groups based on the total score.

OP risk and bone density status were evaluated using the following methods: BMD at the lumbar spine and hip was measured by dual-energy X-ray absorptiometry (DXA), and T-scores were calculated; concurrently, the Fracture Risk Assessment Tool (FRAX®) was utilized to calculate the 10-year probability of major OF and hip fractures.

BMD at the lumbar spine and hip was measured by dual-energy X-ray absorptiometry (DXA). Both T-scores (comparison to young adult peak bone mass) and *Z*-scores (comparison to age-, sex-, and ethnicity-matched references) were calculated and recorded.

### 2.3. Blood sample collection and biomarker measurement

Fasting peripheral venous blood samples were collected from each participant in the morning. The samples were processed within 2 hours of collection. Serum was separated by centrifugation and aliquoted into cryogenic vials. All samples were stored at −80°C until batch analysis to ensure stability of the biomarkers.

The serum levels of BTMs, including PINP, β-CTX, and N-MID, were quantitatively determined using electrochemiluminescence immunoassay on a Roche Cobas e analyzer, following the manufacturer’s instructions. Serum E2 levels were measured using a competitive chemiluminescent immunoassay.

### 2.4. Statistical analysis

Descriptive statistics were used to summarize patient characteristics. Continuous variables are presented as mean ± standard deviation, while categorical variables are expressed as frequency and percentage. Based on the distribution of the data, independent samples *t*-test was employed for group comparisons.

Spearman’s correlation coefficient was used to analyze the correlations between depression severity (HAMD-17 score) and BMD values as well as FRAX fracture risk scores. The choice of this specific method was based on the results of normality tests. A 2-sided *P*-value < .05 was considered statistically significant.

To evaluate the potential impact of depression severity on OP and fracture risk, we established 2 multivariate regression models: multivariate logistic regression analysis was used to assess the influence of depression severity on the risk of developing OP, while multivariate Cox proportional hazards (PH) regression model was employed to evaluate the impact of depression severity on subsequent fracture risk. Both models were adjusted for potential confounding factors, including age, BMI, years since menopause, and smoking history.

To further investigate the stability and generalizability of the association between depression severity and bone health, we conducted subgroup analyses based on age (grouped by median), years since menopause (≤5 years vs >5 years), and the presence of diabetes comorbidity.

The validity of the assumptions underlying the primary statistical models was assessed prior to analysis. For the multivariate logistic regression models, the assumptions of linearity in the logit for continuous covariates and the absence of multicollinearity were examined. Linearity was assessed using the Box–Tidwell procedure, and no significant deviations were found. Multicollinearity was evaluated by calculating the variance inflation factor (VIF) for all independent variables; all VIF values were below 2.0, indicating no substantial multicollinearity.

For the multivariate Cox PHs regression models, the PHs assumption – that the hazard ratio between any 2 subjects is constant over time – was rigorously assessed using Schoenfeld residuals. This involved a statistical test for each covariate and a global test for the overall model, with results presented in Table S1, Supplemental Digital Content, https://links.lww.com/MD/R443. The global Grambsch–Therneau test yielded χ^2^ = 4.62, *df* = 5, *P* = .47, confirming no violation of the PH assumption. Thus, the hazard ratios can be considered constant across the follow-up period.

For the multivariate logistic regression models, the following key assumptions were rigorously assessed prior to interpreting the final model:

Independence of observations: This was ensured by the study design, as each participant was recruited and measured independently.

Linearity in the logit for continuous predictors: The assumption of a linear relationship between continuous independent variables (age, BMI, years since menopause) and the log-odds of osteoporosis was tested using the Box–Tidwell procedure. The results are presented in Table S2, Supplemental Digital Content, https://links.lww.com/MD/R443.

Absence of multicollinearity: This was assessed by calculating the tolerance and VIF for each predictor in the full model. A VIF value exceeding 10 (or Tolerance below 0.1) is often considered indicative of severe multicollinearity. The results are presented in Table S3, Supplemental Digital Content, https://links.lww.com/MD/R443.

Lack of overly influential outliers: The influence of individual data points on the model coefficients and predictions was assessed using Cook’s distance and standardized deviance residuals. Observations with a Cook’s distance >1, or standardized residuals with an absolute value >3, are commonly flagged. The summary of influential case diagnostics is presented in Table S4, Supplemental Digital Content, https://links.lww.com/MD/R443.

Furthermore, the overall goodness-of-fit of the final logistic regression model (model 2) was evaluated using the Hosmer–Lemeshow test, classification accuracy metrics, and the area under the receiver operating characteristic curve (AUC). These results are consolidated in Table S5, Supplemental Digital Content, https://links.lww.com/MD/R443.

All statistical analyses were performed using SPSS statistics version 25.0 (IBM Corporation, Armonk).

### 2.5. Post-hoc power analysis

To assess whether the enrolled sample size provided adequate statistical power to detect the observed associations, a post hoc power analysis was conducted using G*Power software (version 3.1.9.7). Given the primary objective of evaluating the association between depression severity (as a 3-level categorical variable) and the prevalence of osteoporosis (a binary outcome), the analysis was based on the chi-square test for trend (proportions). The input parameters were derived from our observed data: the sample size of N = 245, an effect size (w) of 0.30 (calculated from the observed proportion differences in osteoporosis prevalence across depression severity groups: 21.4%, 31.4%, and 60.0%), a significance level (α) of 0.05, and 3 groups (*df* = 1 for trend). This calculation yielded a statistical power of 96.8%. Furthermore, for the key continuous outcome of lumbar spine BMD, a post hoc analysis for 1-way ANOVA across the 3 depression groups, with an observed effect size (f) of 0.40 (derived from the group means and standard deviations), a sample size of 245, and α = 0.05, yielded a power of 99.5%. These results indicate that the study sample provided excellent statistical power (>80%) to detect the clinically meaningful associations reported in this cohort, supporting the robustness and potential generalizability of the findings.

## 3. Results

### 3.1. Clinical characteristics

This study included a total of 245 postmenopausal women. The baseline demographic and clinical characteristics of the study participants are presented in Table 1. The mean age of the subjects was 62.4 ± 6.8 years, and the mean age at natural menopause was 50.1 ± 3.5 years. The mean BMI for the entire cohort was 24.2 ± 3.6 kg/m^2^, which falls within the upper range of normal. Regarding lifestyle and medical history, 49 individuals (20.0%) had a history of smoking, and 28 (11.4%) had a previous history of nontraumatic fracture. In terms of comorbidities, hypertension was the most prevalent, affecting 102 participants (41.6%), followed by type 2 diabetes mellitus (T2DM) in 57 patients (23.3%) and osteoarthritis in 45 patients (18.4%).

**Table 1:** Clinical characteristics of the study population.

Characteristic	Value (n = 245, mean ± SD or n [%])
Demographic indicators	
Age (yr)	62.4 ± 6.8
Age at menopause (yr)	50.1 ± 3.5
BMI (kg/m^2^)	24.2 ± 3.6
Lifestyle and medical history	
Smoking history, n (%)	49 (20.0%)
History of previous fracture, n (%)	28 (11.4%)
Common cComorbidities, n (%)	
Hypertension	102 (41.6%)
Type 2 diabetes mellitus	57 (23.3%)
Osteoarthritis	45 (18.4%)

BMI = body mass index, SD =standard deviation.

### 3.2. Correlation between depressive severity and comprehensive bone health indicators

Patients were stratified into 3 groups based on depression severity using the HAMD-17 scale. Compared to the no/mild depression group, both moderate and severe depression groups demonstrated progressively worse skeletal health parameters. The severe depression group showed significantly lower lumbar spine BMD (0.79 ± 0.12 g/cm^2^ vs 0.95 ± 0.11 g/cm^2^, *P* < .001) and hip BMD (0.75 ± 0.11 g/cm^2^ vs 0.89 ± 0.10 g/cm^2^, *P* = .023), along with markedly elevated BTMs including PINP (76.3 ± 23.1 μg/L vs 45.1 ± 14.2 μg/L, *P* < .001) and β-CTX (0.60 ± 0.17 ng/L vs 0.35 ± 0.10 ng/L, *P* < .001). Furthermore, the severe depression group exhibited significantly reduced E2 levels (9.2 ± 3.5 pg/mL vs 15.8 ± 5.0 pg/mL, *P* = .018) and substantially higher prevalence rates of both OP (60.0% vs 21.4%, *P* < .001) and OF (87.5% vs 18.8%, *P* < .001).

A similar trend was observed for age-matched *Z*-scores. Both lumbar spine and hip *Z*-scores showed a significant inverse correlation with depression severity (*P* for trend <.001). The severe depression group had markedly lower lumbar spine *Z*-scores (−1.9 ± 1.1) and hip *Z*-scores (−1.6 ± 1.0) compared to the no/mild depression group (−0.8 ± 1.0 and −0.6 ± 0.9, respectively; all *P* < .01). This indicates that the detrimental association between depression and BMD persists even after accounting for the expected age-related decline in bone mass. Detailed comparisons of skeletal health indicators across depression severity groups are presented in Table 2.

**Table 2:** Comparison of skeletal health indicators across depression severity groups.

Indicator	No/mild depression	Moderate depression	Severe depression	*P*-value	OR (95% CI) ^+^
Sample information	n = 140 (57.1%)	n = 70 (28.6%)	n = 35 (14.3%)		(Reference)
Bone density indicators					
Lumbar spine BMD (g/cm^2^)	0.95 ± 0.11	0.87 ± 0.09	0.79 ± 0.12	<.001	0.55 (0.40–0.76)
Hip BMD (g/cm^2^)	0.89 ± 0.10	0.82 ± 0.08	0.75 ± 0.11	.023	0.68 (0.52–0.89)
BTMs					
PINP (μg/L)	45.1 ± 14.2	58.7 ± 19.5 a	76.3 ± 23.1 ab	<.001	1.18 (1.06–1.31)
β-CTX (ng/L)	0.35 ± 0.10	0.46 ± 0.14 a	0.60 ± 0.17 ab	<.001	1.25 (1.08–1.45)
N-MID (ng/mL)	19.1 ± 5.8	22.5 ± 6.3 a	26.8 ± 7.4 ab	.034	1.15 (1.02–1.30)
Bone metabolism and hormones					
E2 (pg/mL)	15.8 ± 5.0	12.1 ± 4.3 a	9.2 ± 3.5 ab	.018	0.78 (0.65–0.94)
Disease and risk assessment					
OP prevalence	21.4% (30/140)	31.4% (22/70)	60.0% (21/35)	<.001	2.85 (2.12–3.82)
Osteoporotic fracture prevalence	18.8% (21/112)	41.2% (35/85) a	87.5% (42/48) ab	<0.001	3.42 (2.58–4.53)
10-year major osteoporotic fracture risk (FRAX%)	5.8 ± 2.1	8.9 ± 3.0	13.5 ± 4.2	.041	1.38 (1.24–1.53)
Bone ddensity indicators					
Lumbar spine BMD (g/cm^2^)	0.95 ± 0.11	0.87 ± 0.09	0.79 ± 0.12	<.001	0.55 (0.40–0.76)
Lumbar spine *Z*-score	−0.8 ± 1.0	−1.3 ± 0.9	−1.9 ± 1.1	<.001	0.62 (0.48–0.80)
Hip BMD (g/cm^2^)	0.89 ± 0.10	0.82 ± 0.08	0.75 ± 0.11	.023	0.68 (0.52–0.89)
Hip *Z*-score	−0.6 ± 0.9	−1.1 ± 0.8	−1.6 ± 1.0	.005	0.70 (0.55–0.89)

Data are presented as mean ± standard deviation or % (n/N). *P*-value for trend across groups was determined by 1-way ANOVA for continuous variables or chi-square test for prevalence rates. “a” Significantly different from the no/mild depression group (*P* < .05). “b” Significantly different from the moderate depression group (*P* < .05).

β-CTX = beta-crosslaps (C-terminal telopeptide of type I collagen), ANOVA = analysis of variance, BMD = bone mineral density, BTM = bone turnover marker, E2 = estradiol, FRAX = Fracture Risk Assessment Tool, N-MID = N-terminal mid-fragment of osteocalcin, OP = osteoporosis, PINP = procollagen type I N-terminal propeptide.

### 3.3. The effect of depressive severity on the risk of OP

Multivariate logistic regression analysis was performed to assess the association between depression severity and OP risk. In the unadjusted model (model 1), both moderate depression (OR = 1.75, 95% CI: 1.42–2.15, *P* < .001) and severe depression (OR = 3.10, 95% CI: 2.45–3.92, *P* < .001) were significantly associated with increased OP risk compared to the no/mild depression reference group. After full adjustment for covariates including age, BMI, years since menopause, and smoking history (model 2), these associations remained statistically significant, though slightly attenuated. Specifically, patients with moderate depression showed 1.68-fold increased odds (95% CI: 1.35–2.08, *P* < .001), while those with severe depression demonstrated 2.85-fold increased odds (95% CI: 2.21–3.67, *P* < .001) of developing OP. Among the adjusted covariates, advancing age (OR = 1.08 per year, *P* = .001), longer postmenopausal duration (OR = 1.06 per year, *P* = .015), and smoking history (OR = 1.52, *P* = .011) were identified as significant risk factors, while higher BMI served as a protective factor (OR = 0.92 per kg/m^2^, *P* = .007). Detailed results of the multivariate logistic regression analysis are presented in Table 3.

**Table 3:** Multivariate logistic regression analysis of depression severity and OP risk.

Variable	Model 1 (unadjusted)	Model 2 (fully adjusted)	OR (95% CI)	*P*-value
	OR (95% CI)	*P*-value		
Depression severity				
No/mild depression	1.00 (Reference)	–	1.00 (Reference)	–
Moderate depression	1.75 (1.42–2.15)	<.001	1.68 (1.35–2.08)	<.001
Severe depression	3.10 (2.45–3.92)	<.001	2.85 (2.21–3.67)	<.001
Covariates				
Age (per 1-yr increase)	Not included	–	1.08 (1.03–1.13)	.001
BMI (per 1 kg/m^2^ increase)	Not included	–	0.92 (0.87–0.98)	.007
Years since menopause (per 1-yr increase)	Not included	–	1.06 (1.01–1.11)	.015
Smoking history (yes vs no)	Not included	–	1.52 (1.10–2.10)	.011

Model 2 was adjusted for all covariates listed in the table (age, body mass index, years since menopause, and smoking history).

BMI = body mass index, CI =confidence interval, OP = osteoporosis, OR = odds ratio.

The validity of the multivariate logistic regression model (model 2) was confirmed through comprehensive diagnostic testing. All key assumptions were upheld: the linearity assumption for continuous predictors was met (Box–Tidwell procedure, all *P* > .05; Table S1, Supplemental Digital Content, https://links.lww.com/MD/R443); no substantial multicollinearity was detected among predictors (all VIFs < 2; Table S2, Supplemental Digital Content, https://links.lww.com/MD/R443); and no influential outliers or high-leverage points were identified (maximum Cook’s distance = 0.118; Table S3, Supplemental Digital Content, https://links.lww.com/MD/R443). The model demonstrated adequate overall fit (Hosmer–Lemeshow test: *P* = .520) and excellent discriminatory power for osteoporosis (AUC = 0.829; Table S4, Supplemental Digital Content, https://links.lww.com/MD/R443). These results affirm the robustness and reliability of the reported odds ratios.

### 3.4. Influence of depressive severity on risk of osteoporotic fracture

The underlying PHs assumption for the Cox regression model was validated. Statistical testing based on Schoenfeld residuals revealed no significant violations for any covariate or the model globally (all *P* > .05; see Table S5, Supplemental Digital Content, https://links.lww.com/MD/R443). Multivariate Cox regression analysis revealed a significant dose-response relationship between depression severity and fracture risk. In the unadjusted model, patients with moderate and severe depression showed 2.05-fold (95% CI: 1.58–2.66, *P* < .001) and 3.75-fold (95% CI: 2.78–5.06, *P* < .001) increased fracture risks, respectively, compared to the no/mild depression reference group. After full adjustment for age, BMI, years since menopause, smoking history, and baseline BMD T-score, these associations remained statistically significant though attenuated, with moderate and severe depression groups demonstrating 1.82-fold (95% CI: 1.39–2.38, *P* < .001) and 2.90-fold (95% CI: 2.10–4.00, *P* < .001) increased risks, respectively. Among the adjusted covariates, baseline BMD T-score emerged as the strongest predictor (HR = 1.65 per 1 SD decrease, *P* < .001), followed by advancing age (HR = 1.05 per year, *P* = .008), longer postmenopausal duration (HR = 1.04 per year, *P* = .043), and smoking history (HR = 1.45, *P* = .023), while higher BMI served as a protective factor (HR = 0.90 per kg/m^2^, *P* < .001). Detailed results of the multivariate Cox regression analysis are presented in Table 4.

**Table 4:** Multivariate Cox regression analysis of the association between depression severity and risk of subsequent fractures.

Variable	Model 1 (unadjusted)	Model 2 (fully adjusted)	HR (95% CI)	*P*-value
	HR (95% CI)	*P*-value		
Depression severity				
No/mild depression	1.00 (Reference)	–	1.00 (Reference)	–
Moderate depression	2.05 (1.58–2.66)	<.001	1.82 (1.39–2.38)	<.001
Severe depression	3.75 (2.78–5.06)	<.001	2.90 (2.10–4.00)	<.001
Covariates				
Age (per 1-yr increase)	Not included	–	1.05 (1.01–1.09)	.008
BMI (per 1 kg/m^2^ increase)	Not included	–	0.90 (0.85–0.95)	<.001
Years since menopause (per 1-year increase)	Not included	–	1.04 (1.00–1.08)	.043
Smoking history (yes vs no)	Not included	–	1.45 (1.05–2.00)	.023
Baseline BMD T-score (per 1 SD decrease)	Not included	–	1.65 (1.40–1.95)	<.001

Model 2 was adjusted for all covariates listed in the table (age, BMI, years since menopause, smoking history, and baseline BMD T-score). All variables were independent predictors of osteoporotic fracture in the fully adjusted model.

BMD = bone mineral density, BMI = body mass index, CI = confidence interval, SD = standard deviation.

### 3.5. Influence of depressive severity on risk of osteoporotic fracture

Subgroup analyses revealed significant variations in the association between depression severity and hip BMD across different patient populations. In women with longer postmenopausal duration (>5 years), depression severity demonstrated a stronger negative correlation with BMD (OR = 0.93, 95% CI: 0.91–0.94, *P* < .001) compared to those within 5 years of menopause (OR = 0.95, 95% CI: 0.92–0.97, *P* < .001), with the interaction analysis reaching statistical significance (*P* = .043).

Similarly, among patients with comorbid diabetes, the inverse association between depression severity and BMD was particularly pronounced (OR = 0.91, 95% CI: 0.89–0.93, *P* < .001), significantly stronger than that observed in nondiabetic patients (OR = 0.94, 95% CI: 0.93–0.95, *P* < .001; *P* = .004).

However, no significant differential effect was observed between age subgroups. Both younger (≤62 years) and older (>62 years) patients showed comparable inverse associations between depression severity and BMD (OR = 0.93 vs OR = 0.94, *P* = .215).

These subgroup analyses highlight depression severity as a significant predictor of reduced BMD, particularly in patients with longer postmenopausal duration and those with comorbid diabetes. Detailed results of the subgroup analysis are presented in Table 5.

**Table 5:** Subgroup analysis of the association between depression severity and bone mineral density.

Subgroup	Number of patients (total 245)	OR (95% CI)*	*P*-value	*P* for interaction
Age				.215
≤62 yr	128	0.93 (0.91, 0.95)	<.001	
>62 yr	117	0.94 (0.92, 0.96)	<.001	
Years since menopause				.043
≤5 yr	85	0.95 (0.92, 0.97)	<.001	
>5 yr	160	0.93 (0.91, 0.94)	<.001	
Comorbid diabetes				.004
Yes	57	0.91 (0.89, 0.93)	<.001	
No	188	0.94 (0.93, 0.95)	<.001	

BMD = bone mineral density, CI =confidence interval, OR = odds ratio.

* The “OR” values presented are approximate effect sizes derived from exponentiated β-coefficients (e^β^) obtained from linear regression models analyzing the association between depression severity and hip bone mineral density within each subgroup. An OR < 1 indicates a negative association (i.e., more severe depression is associated with lower BMD).

## 4. Discussion

Our study makes several distinct contributions to the advancement of research on the intersection of mental and skeletal health in postmenopausal women. First, it moves beyond the established epidemiological link by establishing a clear, dose-response relationship between depression severity (quantified by HAMD-17) and a comprehensive panel of bone health indices, including BMD, bone turnover markers (PINP, β-CTX, N-MID), and estradiol levels. This granular analysis provides stronger mechanistic support for a causal pathway than binary comparisons of depression presence/absence. Second, it prospectively validates depression severity as an independent and significant predictor of incident osteoporotic fractures, even after adjusting for baseline BMD and other established risk factors. This finding addresses a critical gap in prognostic research and positions depression assessment as a tangible component of fracture risk stratification. Third, our subgroup analyses uncover high-risk patient phenotypes, identifying women with longer postmenopausal duration (>5 years) and those with comorbid diabetes as subgroups in whom the detrimental skeletal effects of depression are markedly amplified. This moves the field from a general association towards personalized risk profiling. Collectively, these findings shift the paradigm by framing depression not merely as a comorbidity but as a modifiable, independent risk factor and potential therapeutic target within the integrated management of postmenopausal osteoporosis.

Our findings align with and substantially extend the existing body of epidemiological and clinical literature on the depression-osteoporosis link. Previous large-scale epidemiological studies, such as the meta-analysis by Funakoshi et al (2024),^[9]^ have established a significant association between depression and increased fracture risk in older adults. Our results confirm this association in the specific high-risk demographic of postmenopausal women. However, much of the prior literature has treated depression as a binary variable. Our study advances this understanding by demonstrating a clear dose-response relationship between depression severity (HAMD-17 score) and both bone density decline and fracture incidence, a gradient effect that has been less frequently quantified in previous cohort studies.

Furthermore, while prior clinical studies have often focused on BMD as the primary outcome, our integrated assessment of bone turnover markers (PINP, β-CTX) and estradiol provides a more granular, mechanistic link that supports proposed biological pathways, such as HPA axis dysregulation and chronic inflammation. This multi-parameter approach offers stronger clinical evidence for the pathophysiological interplay than studies relying solely on BMD or fracture registries.

Notably, our identification of postmenopausal duration (>5 years) and comorbid diabetes as effect modifiers that significantly amplify the depression-bone loss association addresses a gap in the literature. While diabetes is a known risk factor for osteoporosis, and longer estrogen deficiency is a driver of bone loss,^[1]^ their synergistic interaction with depression severity for fracture risk has been less clearly delineated. Our subgroup findings thus contribute to a more nuanced, personalized risk stratification model, moving beyond the general population-level risks reported in earlier epidemiological works.

The pathophysiological mechanisms underlying the depression-OP connection appear multifactorial and complex. Depression induces chronic activation of the HPA axis, leading to sustained cortisol elevation that directly inhibits osteoblast activity and promotes osteoclastogenesis through RANKL activation.^[11]^ Concurrently, depression is characterized by a pro-inflammatory state, with elevated levels of IL-6, TNF-α, and other cytokines that further activate bone resorption pathways.^[12]^ Our findings of significantly altered BTMs (PINP, β-CTX, N-MID) and reduced estrogen levels in depressed women provide mechanistic support for these pathways. The gut-bone axis may represent another important mechanism, as depression-related gut microbiota alterations affect calcium absorption and bone metabolism through immune modulation.^[13]^

Notably, our subgroup analyses revealed particularly pronounced associations in women with longer postmenopausal duration (>5 years) and those with comorbid diabetes. The synergistic effect between prolonged estrogen deficiency and depression-related pathophysiology suggests a cumulative detrimental impact on bone metabolism.^[14]^ The strong association in diabetic patients aligns with emerging evidence of shared biological pathways, including chronic inflammation, oxidative stress, and advanced glycation end-product accumulation, which collectively accelerate bone deterioration.^[15]^

Behavioral factors significantly contribute to this relationship. Depression is associated with poorer nutrition, particularly inadequate calcium and vitamin D intake,^[16]^ reduced physical activity,^[17]^ decreased sun exposure, and lower adherence to OP medications. These factors collectively create an environment conducive to accelerated bone loss and increased fracture risk.

The clinical implications of our findings are substantial. Current evidence supports integrating routine depression screening into OP risk assessment protocols for postmenopausal women.^[18]^ Patients with moderate to severe depression warrant intensified bone health monitoring, including regular BMD assessments and fracture risk evaluation using FRAX®.^[19]^ An integrated treatment approach addressing both mental and skeletal health is essential. Selective serotonin reuptake inhibitors may have varying effects on bone metabolism, requiring careful consideration in treatment selection.^[20]^ Non-pharmacological interventions such as exercise programs combining weight-bearing and resistance training can simultaneously benefit both depression and bone health.^[21]^

Emerging evidence suggests that certain antidepressants, particularly bupropion, may have neutral or even protective effects on bone metabolism compared to Selective serotonin reuptake inhibitors.^[22]^ Furthermore, the timing of antidepressant initiation relative to menopause transition may influence their skeletal effects.^[23]^ Future research should explore whether successful antidepressant therapy can attenuate bone loss and reduce fracture risk.

Despite these significant findings, several limitations merit consideration. The mixed cross-sectional and prospective design, while providing valuable insights, limits definitive causal inference regarding the temporal relationship between depression and bone loss. Residual confounding from unmeasured factors such as detailed dietary assessments, physical activity levels, and vitamin D status may persist. Additionally, the single-center nature of our study may affect generalizability to broader populations. And, our fracture assessment relied on radiographic confirmation of clinically symptomatic or suspected fractures, and did not routinely perform vertebral fracture assessment via dual-energy X-ray absorptiometry. Consequently, asymptomatic vertebral fractures, which are common and contribute significantly to morbidity and future fracture risk, may have been under-detected in our cohort. Additionally, our bone health evaluation was based on areal BMD measured by DXA, which is the current clinical gold standard. We did not assess trabecular bone score, a texture parameter derived from DXA images that provides complementary information on bone microarchitecture and fracture risk independent of BMD. Future studies incorporating both vertebral fracture assessment and trabecular bone score alongside traditional BMD and clinical assessment would provide a more comprehensive and sensitive evaluation of skeletal fragility in depressed postmenopausal women, potentially strengthening the observed associations and refining risk prediction.

Future research should include longitudinal studies with repeated measurements of both depression severity and bone health parameters to establish causal pathways. Multicenter studies with diverse populations would enhance the generalizability of findings. Investigation into the effects of different antidepressant classes on bone metabolism, and whether successful treatment of depression can modify fracture risk, represents another crucial direction for future research. Exploration of novel interventions that simultaneously target both mental health and bone health pathways could lead to more effective integrated treatment strategies.

## 5. Conclusion

In conclusion, this study provides compelling evidence that depression severity independently predicts OP risk and subsequent fractures in postmenopausal women, with particularly strong effects in those with longer postmenopausal duration and comorbid diabetes. These findings underscore the necessity of incorporating mental health assessment into bone health management protocols and call for integrated treatment strategies addressing both conditions simultaneously. The recognition of depression as a significant risk factor for OP represents an important advancement in our approach to fracture prevention in postmenopausal women.

## Author contributions

**Conceptualization:** Yichao Qiu.

**Data curation:** Yichao Qiu.

**Formal analysis:** Xiaoqin Qian, Haifang Sun.

**Investigation:** Lina Yu.

**Methodology:** Jiande Chen.

**Validation:** Xiang Chen.

**Writing – original draft:** Yichao Qiu.

**Writing – review & editing:** Yichao Qiu.

## Supplementary Material


