# Supplementary material for: The relationship between the severity of depression in postmenopausal women and the incidence of osteoporosis and subsequent fractures: A cross-sectional observational study
Source: Medicine (Baltimore). 2026 Feb 28;105(9):e47750. doi: 10.1097/MD.0000000000047750 (PMC12956237; doi:10.1097/MD.0000000000047750)
Supplement: Supplementary file 1 [file medi-105-e47750-s001.docx]

**Supplementary Tables**

**Supplementary Table S1. Assessment of Linearity Assumption for Continuous Predictors Using the Box-Tidwell Procedure.**

| Continuous Predictor | Interaction Term | Coefficient (β) | Standard Error | Wald χ² | p-value | Linearity Assumption |
| --- | --- | --- | --- | --- | --- | --- |
| Age | Age * ln(Age) | 0.037 | 0.045 | 0.676 | 0.411 | Upheld |
| Body Mass Index (BMI) | BMI * ln(BMI) | -0.021 | 0.039 | 0.290 | 0.590 | Upheld |
| Years Since Menopause | YSM * ln(YSM) | 0.058 | 0.048 | 1.460 | 0.227 | Upheld |

**Supplementary Table S2. Assessment of Multicollinearity Among Independent Variables in the Final Logistic Regression Model (Model 2).**

| Predictor | Tolerance | VIF | Assessment |
| --- | --- | --- | --- |
| Age (years) | 0.832 | 1.202 | No multicollinearity |
| Body Mass Index (BMI, kg/m²) | 0.901 | 1.110 | No multicollinearity |
| Years Since Menopause (YSM) | 0.794 | 1.259 | No multicollinearity |
| Smoking History (Yes vs. No) | 0.958 | 1.044 | No multicollinearity |
| Depression Severity (Reference: No/Mild) |  |  |  |
| Moderate Depression | 0.565 | 1.770 | No multicollinearity |
| Severe Depression | 0.523 | 1.912 | No multicollinearity |

Note: All VIF values are well below the conservative threshold of 5 and far below 10, indicating no substantial multicollinearity among the predictors in the model.

**Supplementary Table S3. Diagnostic Summary for Influential Observations and Outliers in the Logistic Regression Model.**

| Diagnostic Measure | Criterion for Concern | Result in Current Model | Conclusion | |  |
| --- | --- | --- | --- | --- | --- |
| Cook's Distance (D) | D > 1.0 | Maximum D = 0.118 | No influential cases detected. |  |  |
|  |  | Number of cases with D > 0.5: 0 |  |  |  |
| Standardized Deviance Residuals |  | Absolute Value > 3.0 | Maximum Absolute Value = 2.41 | No extreme outliers detected. |  |
|  |  | Number of cases with \|Residual\| > 3: 0 |  |  |  |
| Leverage (Hat Values) | > 2×(k+1)/n * | Maximum Leverage = 0.089 | No high-leverage points detected. |  |  |
|  | (where k = number of predictors, n = sample size) | Threshold ≈ 0.057 |  |  |  |

Note: * A common rule-of-thumb threshold for high leverage is 2×(k+1)/n, where k is the number of predictors (6) and n is the sample size (245). The calculated threshold is 2×(6+1)/245 ≈ 0.057. All observed leverage values were below or near this threshold.

**Supplementary Table S4. Goodness-of-Fit and Predictive Performance of the Final Multivariate Logistic Regression Model (Model 2).**

| Test / Metric | Value (95% CI if applicable) | Interpretation |
| --- | --- | --- |
| Hosmer-Lemeshow Goodness-of-Fit Test | χ²(8) = 7.15, p = 0.520 | The model fits the data adequately (p > 0.05 indicates no significant lack of fit). |
| Cox & Snell R² | 0.281 | Pseudo R-squared indicating proportion of variance explained. |
| Nagelkerke R² | 0.414 | Adjusted pseudo R-squared, often more interpretable. |
| Overall Classification Accuracy | 78.4% | Percentage of cases correctly classified by the model. |
| Sensitivity (True Positive Rate) | 72.6% | Model's ability to correctly identify osteoporosis cases. |
| Specificity (True Negative Rate) | 81.9% | Model's ability to correctly identify non-osteoporosis cases. |
| Area Under the ROC Curve (AUC) | 0.829 (0.783 – 0.874) | Excellent discriminatory ability (AUC > 0.8). |

**Supplementary Table S5. Proportional-hazards assumption check based on scaled Schoenfeld residuals**

| Covariate |  |  |  | χ² (Chi-square) | df | p-value | Conclusion (α=0.05) |
| --- | --- | --- | --- | --- | --- | --- | --- |
| Depression severity (per 1-level ↑) | 0.08 | 0.007 | 0.032 | 0.05 | 1 | 0.83 | Assumption upheld |
| Age (per 1-year ↑) | 0.11 | 0.004 | 0.006 | 0.44 | 1 | 0.51 | Assumption upheld |
| BMI (per 1 kg·m⁻² ↑) | –0.06 | –0.005 | 0.011 | 0.20 | 1 | 0.65 | Assumption upheld |
| Years since menopause (per 1-year ↑) | 0.09 | 0.008 | 0.012 | 0.44 | 1 | 0.51 | Assumption upheld |
| Smoking (Yes vs No) | 0.03 | 0.009 | 0.041 | 0.05 | 1 | 0.82 | Assumption upheld |
| Baseline BMD T-score (per 1 SD ↓) | –0.07 | –0.006 | 0.015 | 0.16 | 1 | 0.69 | Assumption upheld |
| Global test | — | — | — | 4.62 | 5 | 0.47 |  |

Notes: PH assumption was tested using the scaled Schoenfeld residuals. A non-significant p-value (p > 0.05) indicates no evidence against the proportional hazards assumption for that covariate or the model overall.
